# Do Implicit and Explicit Measures of the Sense of Agency Measure the Same Thing?

**DOI:** 10.1371/journal.pone.0110118

**Published:** 2014-10-16

**Authors:** John A. Dewey, Günther Knoblich

**Affiliations:** Department of Cognitive Science, Central European University, Budapest, Hungary; Max Planck Institute for Human Cognitive and Brain Sciences, Germany

## Abstract

The sense of agency (SoA) refers to perceived causality of the self, i.e. the feeling of causing something to happen. The SoA has been probed using a variety of explicit and implicit measures. Explicit measures include rating scales and questionnaires. Implicit measures, which include sensory attenuation and temporal binding, use perceptual differences between self- and externally generated stimuli as measures of the SoA. In the present study, we investigated whether the different measures tap into the same self-attribution processes by determining whether individual differences on implicit and explicit measures of SoA are correlated. Participants performed tasks in which they triggered tones via key presses (operant condition) or passively listened to tones triggered by a computer (observational condition). We replicated previously reported effects of sensory attenuation and temporal binding. Surprisingly the two implicit measures of SoA were not significantly correlated with each other, nor did they correlate with the explicit measures of SoA. Our results suggest that some explicit and implicit measures of the SoA may tap into different processes.

## Introduction

The sense of agency (SoA) refers to perceived causality of the self, i.e. the feeling of causing something to happen. To varying degrees, people control what they perceive by performing actions which have predictable effects on their environment. This allows people to distinguish the contingent sensory consequences of voluntary actions, henceforth “action-effects”, from externally generated stimuli. It has been argued that the SoA is not a unitary phenomenon, but rather a compound of basic experiences [1], [2]. The SoA is influenced by a combination of predictive and inferential processes, including (but not limited to) efferent motor signals [3], [4] temporal contiguity between movements and action-effects [5], [6], and prior beliefs about the causal structure of the environment [7], [8].

In laboratory settings, the SoA has been probed using a variety of different measures. We distinguish two general approaches to measuring the SoA, explicit and implicit measures. Explicit measures rely on direct judgments of causal efficacy. Implicit measures use perceptual differences between self- and externally generated action-effects as measures of the SoA. There are two main implicit measures of SoA: sensory attenuation and temporal binding. We begin by reviewing what is known about the processes underlying explicit self-attributions, sensory attenuation, and temporal binding. Most of the literature suggests that there should be a close relationship between implicit and explicit measures of the SoA. But it is not clear so far whether the different measures tap into the same self-attribution processes.

### Processes underlying explicit self-attributions

The simplest way to measure the SoA is to explicitly ask participants to whom or what they attribute an action-effect or other event. For example, several studies have used ratings scales on which participants indicate their agreement with statements of the form “I caused X” on a scale from 1 (not at all) to 99 (absolutely) [5], [6], [9]. A large body of literature indicates that these types of explicit self-attributions are influenced by a combination of predictive and inferential processes. One of the most important cues to self-agency is the predictability of the action-effect. Self-generated visual and auditory action-effects are more likely to be incorrectly attributed to other agents when they are spatially distorted, temporally delayed, or incongruent with expectations acquired during previous learning trials [5], [6], [10]. Action-effect predictions are derived from a combination of motor and perceptual cues, weighted differently depending on the context [11].

Explicit self-attributions are also modulated by inferential processes. For example, in everyday experience spatial and temporal contiguity between movements and subsequent perceptions often suggests a causal relationship even if the action is somewhat novel or unpredictable. A role for inference has also been argued for on the basis of experiments where judgments of authorship were influenced by priming outcomes which the actors did not actually intend to produce [9], [12].

Explicit self-attributions can also be influenced by persistent and long-term individual differences related to cognitive capacities or personality. In social and clinical psychology, there is a long history of investigating the extent to which people believe they can control what happens to them, a concept referred to as locus of control [13]. An individual's locus of control is normally measured via questionnaire, and is scored on scale between internal (they believe they can control their life) and external (they believe their life is controlled by external factors they cannot change). The locus of control concept has been shown to predict a variety of behavioral outcomes in health-related and occupational settings [14], [15]. Whether or not a general disposition to experience oneself as an agent influences implicit measures of SoA such as sensory attenuation or temporal binding is unknown. The answer may depend on the degree to which variability in individuals’ locus of control depends on the same motor and perceptual processes involved in anticipating action-effects.

Schizotypy, or prevalence of unusual thoughts and behaviors collectively associated with schizophrenia, can be another dispositional influence on the SoA. A disordered SoA is one of the diagnostic symptoms of schizophrenia [3], [16] and schizotypal personality traits correlate with deficits in predicting the effects of one's own actions [17]. In relation to implicit measures of the SoA (discussed below), there is some evidence that schizophrenic individuals show larger than normal temporal binding effects, perhaps indicating a “hyper-association” between actions and outcomes which may lead to over-attributing sensory consequences of movements to themselves [18]. Others have found that while the predictive component of temporal binding is absent in schizophrenics, the inferential contribution was stronger [19]. However, it is an open question whether normal variations in schizotypy in non-clinical populations correlate with the SoA.

### Processes underlying temporal binding

Temporal binding refers to a perceived temporal attraction between voluntary movements and action-effects [20]. For example, the subjective delay between a voluntary key press and a tone is judged as shorter than the delay between two externally generated tones [21]. One proposal is that temporal binding comes about through an association of forward motor commands with specific action-effects [22]. When it was initially discovered, it was thought to be specific to intentional actions and dependent on three things: an efferent motor signal, reliable temporal relations, and anticipation of the action-effect. However, these ideas have since been challenged. Recent studies have discovered that temporal binding can occur for both self-generated and observed actions, indicating that efferent motor signals are not strictly necessary [21], [23]. Furthermore, reliable temporal relations also appear to be unnecessary, as temporal binding has been reported in studies which employed unpredictable intervals between movements and action-effects, including at super-second intervals well beyond the brief temporal window in which predictive forward models related to motor planning are thought to operate [21], [24], [25]. An alternative to the forward model account is that temporal binding is a more general principle of causal perception which can be explained by Bayesian principles of judgment under uncertainty [26].

Despite deep controversy over the underlying mechanisms, temporal binding has been used as a proxy for the SoA in several studies [4], [27], [28]. The link between temporal binding and SoA is bolstered by findings that prior agency beliefs influence the strength of the binding effect [7], [29]. Furthermore, certain experimental manipulations which modulate the SoA, such as the congruency of action-effects and the valence of outcomes, may also modulate temporal binding [30], [31], [32], [33]; but see also Desantis et al. [34], who reported that the mere presence of an action was sufficient to induce temporal binding irrespective of the outcome.

At the same time, there are interesting dissociations between temporal binding and explicit agency judgments. For example, explicit agency judgments are usually stronger at shorter intervals between movements and action-effects [6], [30]. By contrast, the relationship between interval length and temporal binding is more complicated. In the original temporal binding study [20], temporal binding was robust at fixed intervals of 250 ms, but decayed at longer intervals of 450 or 600 ms. However, other studies have reported temporal binding at intervals as long as 4 s [24], and in some studies the binding effect is actually stronger at longer intervals [30]. These discrepancies seem to be related to methodological differences. Most of the early temporal binding studies used the Libet Clock method [35], in which a rapidly moving clock hand is shown during trials, and participants report the position of the clock hand at the onset of their action or the subsequent action-effect. This approach is well-suited to capturing the perception of event boundaries. By contrast, studies which measure the perception of temporal intervals more directly (via numerical estimates or asking participants to reproduce the interval between two events) have reported temporal binding at much longer intervals [24],[25].

Explicit agency judgments and temporal binding may also diverge in the context of joint action. Obhi and Hall [36] studied the sense of agency using a task in which one participant initiated a movement while a second person was passively moved along with them. They assessed the sense of agency via both explicit self-report and temporal binding. Interestingly, both participants experienced temporal binding effects, but only the initiator reported an explicit sense of agency. Overall, there is compelling evidence that temporal binding and explicit self-attributions rely on overlapping cues, but the nature of the relationship between the two is uncertain, and it is far from clear under what circumstances temporal binding can be considered to be reliable proxy for the SoA.

### Processes underlying sensory attenuation

Sensory attenuation refers to a reduction in the subjective intensity of self-initiated action-effects. For example, tones triggered by participants' voluntary key presses are judged to be less loud than equivalent computer triggered tones [37], [38] and are associated with a reduced N1 component in electrophysiological studies [39] [40]. A proposed mechanism is that sensory predictions generated by forward models in the motor system make incoming sensory feedback more difficult to discriminate when the feedback matches the prediction [41], [42]. This is supported by the finding that attenuation of the auditory N1 depends on accurate action-effect predictions [43].

Similarly to temporal binding, there is controversy over the necessary preconditions for sensory attenuation, and the possibility of a link between sensory attenuation and the SoA is intriguing but still preliminary. For example, in a study investigating the ERP correlates of sensory attenuation and explicit agency judgments, the authors replicated the previously reported attenuation of the N1 for self-generated tones which were congruent with prior action-effect learning. However, it was a different component (the P3a) that directly reflected the explicit agency judgments [40].

Nonetheless, some investigators have argued that sensory attenuation is a unique property of self-generated action-effects [44]. Along similar lines, Stenner and colleagues recently claimed that “sensory attenuation and agency inference depend on overlapping motoric signals” [45]. As with temporal binding, sensory attenuation is also influenced by prior agency beliefs [8]. This leads us to consider sensory attenuation as a potential implicit measure of SoA. It is an open question whether and to what degree sensory attenuation correlates with temporal binding and explicit agency judgments.

### The present study

Despite growing interest in implicit measures of SoA, relatively few studies have compared explicit and implicit measures of the SoA within the same paradigm. The aim of the present study was to investigate the degree to which individual differences in implicit and explicit measures of SoA are correlated. This has both theoretical and practical implications. On the theoretical side, correlations between measures would be consistent with the view that temporal binding, sensory attenuation, and explicit self-attribution depend on overlapping processes. On the practical side, the strength of these correlations is informative for investigators considering using temporal binding or sensory attenuation as alternatives to explicit self-attributions.

As reviewed above, there is evidence that explicit self-attributions, temporal binding, and sensory attenuation are all influenced by action-effect prediction, and both implicit measures are also modulated by prior agency beliefs. Therefore, we hypothesized that explicit and implicit measures of SoA depend on largely the same processes. This would predict strong correlations among all measures. There are several alternatives however. For example, explicit self-attributions might be more influenced by conscious inferences and less by involuntary predictive processes compared to sensory attenuation and temporal binding. This would predict correlations within explicit and implicit measures, but weaker or no correlations between explicit and implicit measures. In general, non-significant correlations between any two measures would suggest that those measures tap different processes which feed into a multidimensional experience of self-agency.

## Methods

### Participants

Seventy-eight participants (49 females and 29 males, mean age 24.10 (*SD* = 3.22)) were recruited from student organizations in the Budapest area in exchange for small vouchers redeemable at local stores. The required sample size was estimated by an a priori power analysis which aimed for a power level (1–β) of.80 and assumed medium sized correlations (.3) or higher between the different measures of SoA. All participants were fluent in English, Hungarian, or both languages, and were given the option of receiving instructions for the experiment in either language. All participants were naïve to the purpose of the study, and gave informed written consent. The study was approved by the United Ethical Review Committee for Research in Psychology (EPKEB).

### Procedure

The procedure included two computer tasks which measured sensory attenuation and temporal binding. In between blocks, participants gave explicit ratings of their SoA during each task using Likert scales. Stimulus presentation was controlled using MATLAB with the Psychophysics toolbox extension [46]. The order of the sensory attenuation and temporal binding tasks was counterbalanced across participants. At the end of the computerized portion of the experiment, the locus of control and magical ideation questionnaires were administered. The total duration of the experiment was about 45 minutes.

### Sensory attenuation task

The sensory attenuation task was a modification of paradigms previously used to investigate sensory attenuation of auditory action-effects [37], [38]. There were two phases to this task: an acquisition phase and a test phase.

During the acquisition phase participants learned an association between a simple motor behavior and a contingent sensory action-effect. Participants were instructed to use their right index finger to press the ‘0' key on the right number keypad, waiting about 2–5 seconds between key presses. Each key press produced a standard tone (800 Hz, 100 ms) following a 50 ms delay. The tones were presented through external speakers at an arbitrary but consistent volume. Participants repeated this 50 times. It is unclear how many learning trials are required for self-triggered tones to become attenuated, but priming experiments suggest action-effect associations can be acquired after as few as 12 repetitions [47].

**Figure 1:**
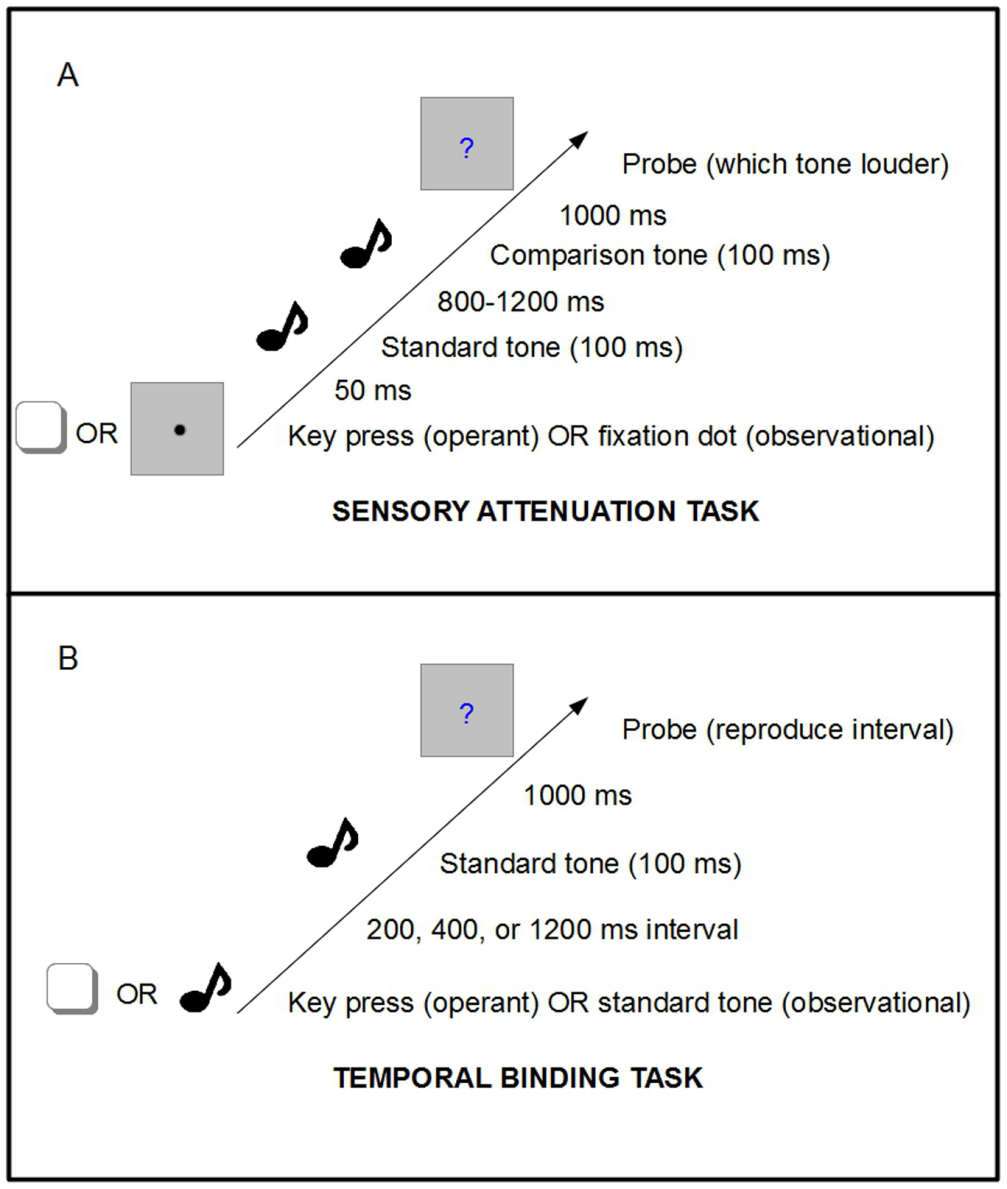
Illustration of test trials in the (A) Sensory attenuation task; and (B) Temporal binding task.

During the test phase, there were two experimental conditions: operant and observational (see Fig. 1A). In the operant condition, participants performed a key press which produced a standard tone following a 50 ms delay. In the observational condition, the computer automatically produced a standard tone without input from the participant. To ensure that the observational condition was temporally predictable, a black fixation dot was shown for 300 ms prior to the standard tone as a warning signal. The fixation dot then disappeared, and the standard tone played 50 ms later. In both conditions, the standard tone was followed by a second tone (the comparison tone) after a random 800–1200 ms delay. Participants were then prompted to perform a two alternative forced choice, judging which of the two tones was louder. The intensity of standard tone was fixed throughout the experiment, while the intensity of the comparison tone was adjusted separately for each condition following a staircase procedure. The staircase procedure converged on the point of subjective equality (PSE) where each tone was judged louder about 50% of the time. In the operant condition, the next trial began as soon as the participant made another key press. In the observational condition, the intertrial interval was 1 s. The operant and observational conditions were blocked, and presented in ABBA order with counterbalancing across participants. There were 30 trials per block, for a total of 120 trials.

Following each block of the operant condition, participants were prompted to rate their SoA on a nine-point scale. The probe read: “During each of the previous trials, you heard two tones. How confident do you feel that your key presses produced the first tone?” Underneath the text was a nine-point scale labeled “not at all” on the far left and “very much” on the far right.

### Temporal binding task

Similarly to the sensory attenuation task, the temporal binding task had an operant and an observational condition (Fig. 1B). In the operant condition, participants performed self-paced key presses with their right index finger, using the ‘0' key on the number keypad. Each key press triggered a standard tone following a delay of 200, 400, or 1200 ms. After the tone, participants were prompted to reproduce the perceived interval between the key press and the onset of the tone by pressing the space bar for a corresponding length of time. In the observational condition participants did not perform key presses, but rather reproduced the interval between two computer triggered tones. The objective interval between the two tones was 200, 400, or 1200 ms.

The operant and observational conditions were blocked, and presented in ABBA order with counterbalancing across participants. Each of the four blocks had 15 repetitions of each delay presented in random order, resulting in 30 repetitions of each delay for each of the operant and observational conditions. There were 45 trials in each block, for a total of 180 trials.

Following each block of the operant condition, participants were prompted to rate their SoA on a nine-point scale. The probe read: “How confident do you feel that your key presses produced the tones?” Underneath the text was a nine-point scale labeled “not at all” on the far left and “very much” on the far right.

### Questionnaires

Two pen-and-paper questionnaires were administered after the computer part of the study, the locus of control questionnaire [13] and the Magical Ideation scale [48]. The Locus of Control scale was used to assess participants' tendencies to feel a sense of control over events in their lives. The Magical Ideation scale assesses participants' tendencies towards magical or superstitious thinking [48], [49] and was used as a measure of individual's placement on a continuum of schizotypy. Two research assistants with high proficiency in both the English and Hungarian languages worked together to provide Hungarian translations of the original questionnaires, as well as translating the instructions for the computerized portion of the experiment.

## Results

### Sensory attenuation

To confirm sensory attenuation of self-initiated tones, the PSE values were submitted to a mixed ANOVA with voluntary action (operant vs. observational) as a within-subjects factor and order of task presentation (SA-TB vs. TB-SA) as a between-subjects factor. Effect sizes are given in generalized eta squared (*η* ^2^G). The raw data for this and all subsequent analyses are available in Tables S1–S3.

**Figure 2:**
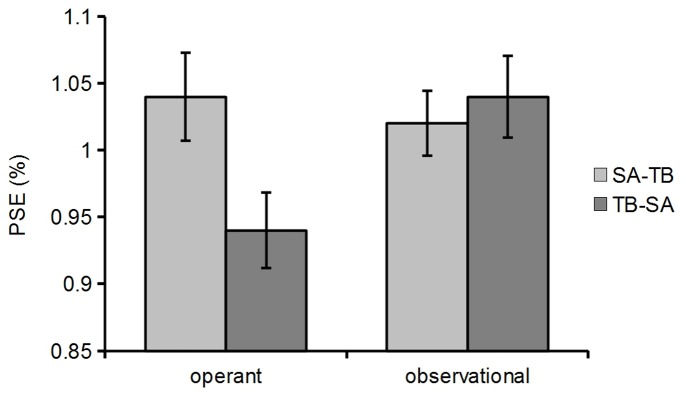
Mean point of subjective equality (PSE) for each condition of the sensory attenuation task. Error bars indicate standard errors of the mean. The ‘1' on the y-axis indicates the point of objective equality where the standard and comparison tones were of equal intensity.

There was no significant main effect of task order [*F*(1, 76) = 1.07, *p* = .30, *η* ^2^G = .01] or voluntary action [*F*(1, 76) = 3.07, *p* = .08, *η* ^2^G = .01], but there was a significant two-way interaction between task order and voluntary action [*F*(1, 76) = 8.48, *p*<.05, *η* ^2^G = .03]. Self-initiated tones were attenuated in the TB-SA group, but not in the SA-TB group (Fig. 2). This indicates that performing the temporal binding task somehow contributed to subsequent sensory attenuation.

### Temporal binding

For each participant, the median reproduced interval for each condition was computed (note: we used medians because they are less susceptible to outliers than means, although in this case it did not affect the results). The intervals were submitted to a mixed ANOVA with voluntary action (operant vs. observational) and temporal delay (200, 400, or 1200 ms) as within-subjects factors, and order of task presentation (SA-TB vs. TB-SA) as a between-subjects factor.

**Figure 3:**
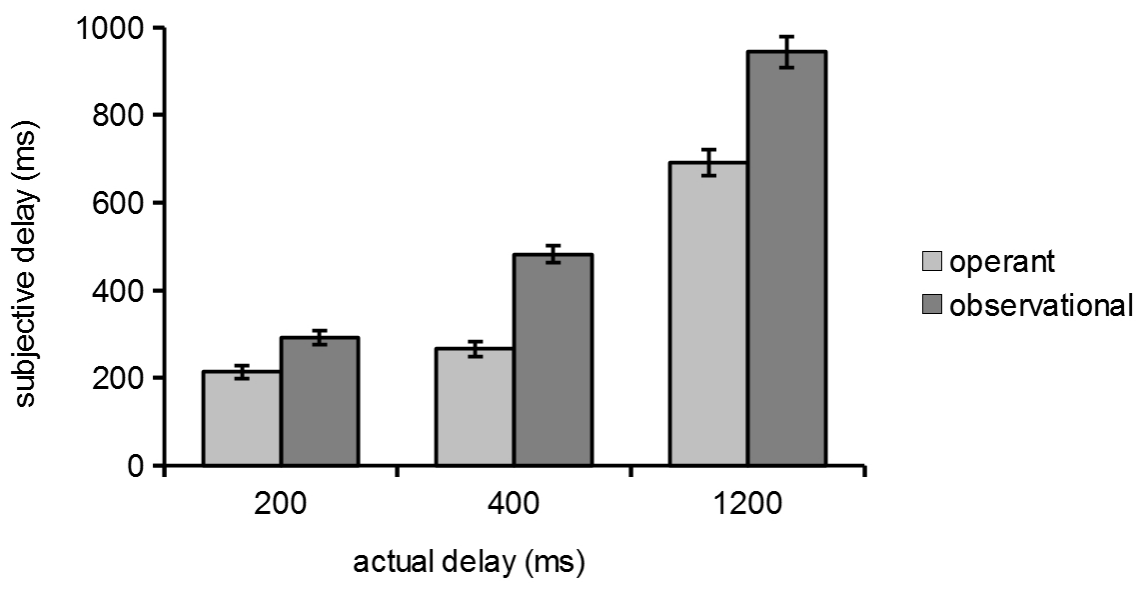
Mean interval reproduction for each condition of the temporal binding task. Error bars indicate standard errors of the mean.

There was no significant main effect of task order [*F*(1, 76) = .19, *p* = .69, *η* ^2^G = .001]. Not surprisingly, there was a significant main effect of temporal delay [*F*(2, 152) = 406.41, *p*<.05, *η* ^2^G = .59], indicating longer interval reproductions at longer delays. More importantly, there was a main effect of voluntary action [*F*(1, 76) = 129.84, *p*<.05, *η* ^2^G = .17], indicating shorter interval reproductions in the operant condition. This is the classic temporal binding effect. There was also a significant two-way interaction between voluntary action and delay [*F*(2, 152) = 49.43, *p*<.05, *η* ^2^G = .03]. The difference between the operant and observational conditions was greater at longer delays (Fig. 3).

### Likert scale ratings

The distribution of Likert scale ratings was left-skewed (i.e. most participants reported medium-to-high confidence they had caused the tones). The ratings were analyzed by applying an aligned rank transform to the data, which were then submitted to a mixed ANOVA with task (SA vs. TB) as a within-subjects factor, and task order (SA-TB vs. TB-SA) as a between-subjects factor. There was no significant effect of task order [*F*(1,76) = .21, *p* = .65, *η* ^2^G = .002]. There was, however, a significant effect of task [*F*(1,76) = 8.13, *p* = .006, *η* ^2^G = .03]. The mean Likert scale rating following blocks of the sensory attenuation task was 6.99, *SEM* = .25, and the mean for the ratings following blocks of the temporal binding task was 5.90, *SEM* = .30. This result was not surprising because the SoA is known to be strongly influenced by temporal contiguity (the delay between key presses and tones was held constant at 50 ms during the sensory attenuation task but alternated between 200, 400, and 1200 ms during temporal binding task). The two-factor interaction was not significant [*F*(1,76) = .09, *p* = .77, *η* ^2^G = .003].

### Questionnaires

The mean score on the Locus of Control scale was 10.99, *SEM* = .50 (intermediate), and the mean score on the Magical Ideation scale was 9.90, *SEM* = .62 (comparable to population norms for college aged populations in the United States [48]). The distribution of scores for both scales was approximately normal.

### Correlations

The focus of this analysis was whether inter-subject differences in the different measures of SoA were correlated with one another. For each individual, a sensory attenuation score and a temporal binding score was computed by subtracting the PSE/reproduced interval for the operant condition from the observational condition. Thus, higher scores corresponded to more sensory attenuation/temporal binding. To correct for false discovery rate, p-values were adjusted using the “fdr” method of Benjamini and Hochberg [50]. Cook's distance was computed for each observation to identify possible outliers, but no data points were found to be unusually influential. All reported correlations are Pearson's R, except for the values in columns seven and eight, which are Spearman's Rho due to the non-normality of the Likert scale ratings. The ninth measure in Table 1 is the difference between the Likert scale ratings collected during the sensory attenuation and temporal binding phases. Since the tones occurred almost immediately following the key presses during the sensory attenuation task, but were delayed by a variable interval in the temporal binding task, this provided a rough indication of how much the explicit SoA was altered by changes in the timing of the tones.

**Table 1:** Correlations between implicit and explicit measure of the SoA, all data (n = 78).

Measure	1	2	3	4	5	6	7	8	9
1. Sensory attenuation	–	.13	.22	.25	−.07	−.10	.17	−.02	.13
2. Temporal binding (200 ms)		−	**.72** *	**.41** *	.04	−.08	.21	.17	.03
3. Temporal binding (400 ms)			−	**.57** *	−.03	−.16	.11	.08	.003
4. Temporal binding (1200 ms)				−	−.16	−.11	.13	.13	−.01
5. Magical Ideation					−	**.35** *	−.11	−.04	−.03
6. Locus of Control						−	−.005	.02	−.04
7. Likert scale rating (SA phase)							−	**.33** *	**.51** *
8. Likert scale rating (TB phase)								−	−**.70** *
9. Likert scale rating (SA – TB phase)									−

*significant at p<.05 (corrected for false discovery rate).

As shown in Table 1, most of the implicit and explicit measures of SoA were not strongly correlated. The strength of temporal binding at each delay was significantly correlated with the other delays, but did not correlate with sensory attenuation or with any other measures. Scores on the Magical Ideation and Locus of Control questionnaires were positively correlated with each other, but not with any other measure. The Likert scale ratings of the SoA collected during the sensory attenuation and temporal binding tasks were correlated with each other, but not with the other measures.

In light of the effect of task order on sensory attenuation, we also computed correlation tables separately for each task order (SA-TB and TB-SA), with the caveat that this reduced the power to detect statistically significant correlations by halving the sample sizes. The correlations for the SA-TB task order is shown in Table 2. The correlations for the TB-SA task order are shown in Table 3.

**Table 2:** Correlations among implicit and explicit measure of the SoA, SA-TB task order (n = 39).

Measure	1	2	3	4	5	6	7	8	9
1. Sensory attenuation	−	.24	.31	**.43** *	−.06	−.06	.33	.07	.14
2. Temporal binding (200 ms)		−	**.79** *	**.44** *	.11	.03	.10	.17	.03
3. Temporal binding (400 ms)			−	**.67** *	.10	.04	.18	.04	.12
4. Temporal binding (1200 ms)				−	.08	.05	.15	.08	.03
5. Magical Ideation					−	.40	−.09	−.13	.12
6. Locus of Control						−	−.02	.15	−.21
7. Likert scale rating (SA phase)							−	**.50** *	.28
8. Likert scale rating (TB phase)								−	−**.71**
9. Likert scale rating (SA – TB phase)									−

*significant at p<.05 (corrected for false discovery rate).

**Table 3:** Correlations among implicit and explicit measure of the SoA, TB-SA task order (N = 39).

Measure	1	2	3	4	5	6	7	8	9
1. Sensory attenuation	−	−.05	.02	.12	−.12	−.16	.06	−.17	.18
2. Temporal binding (200 ms)		−	**.80** *	**.54** *	−.14	−.26	.15	−.11	.04
3. Temporal binding (400 ms)			−	**.65** *	−.17	−.32	.03	.19	−.12
4. Temporal binding (1200 ms)				−	−.25	−.25	.07	.30	−.05
5. Magical Ideation					−	.27	−.11	.13	−.20
6. Locus of Control						−	.06	−.12	.14
7. Likert scale rating (SA phase)							−	.10	**.71**
8. Likert scale rating (TB phase)								−	−**.72**
9. Likert scale rating (SA – TB phase)									−

*significant at p<.05 (corrected for false discovery rate).

Comparing Tables 2 and 3, the only correlations which were consistently significant across both task orders were those between temporal binding scores at the three delays. Sensory attenuation scores significantly correlated with temporal binding at the 1200 ms delay in the SA-TB task order, but not in the TB-SA task order. The Likert scale ratings from the sensory attenuation and temporal binding phases were also correlated in the SA-TB group, but not the TB-SA group.

## Discussion

In recent years an increasing number of investigators been turning to implicit measures, particularly temporal binding, to investigate the SoA [4], [28], [51]. This approach may be attractive for a variety of reasons. For example, implicit measures may reduce the risk that participants will deduce the aims of an experiment and alter or “over think” their responses to satisfy the experimenters. Furthermore there is evidence that temporal binding and sensory attenuation both depend (to varying degrees, and depending on the methodology) on the presence of an efferent motor signal combined with a predictable action-effect (see [42], [52] for reviews of the temporal binding and sensory attenuation literature). However, until now there have been few studies which combined explicit and implicit measures of the SoA in the same paradigm (but see [30] and [53]).

The present study aimed to investigate whether implicit and explicit measures of agency tap into the same processes of self-attribution by asking whether individual differences in implicit and explicit measures of SoA are correlated. We replicated previously reported perceptual differences between self- and externally initiated stimuli. Interestingly, the sensory attenuation effect was only significant in the group of participants who performed the temporal binding task first (TB-SA). It seems plausible that having more experience producing tones accounted for the increased sensory attenuation in the TB-SA group. This suggests that action-effect learning contributes to sensory attenuation. However, since the delays between the key presses and tones varied unpredictably from trial to trial during the temporal binding task, whatever learning occurred did not depend on a reliable temporal relationship between movements and action-effects.

More importantly, neither temporal binding nor sensory attenuation was significantly correlated with explicit measures of the SoA, nor with each other. The only exception was a positive correlation between sensory attenuation and temporal binding at the 1200 ms delay in the SA-TB task order. Unfortunately this difference does not lend itself to a clear interpretation, which leads us to suspect a simple Type I error. Alternatively, this pattern might be explained if sensory attenuation scores early in the experiment (for those in the SA-TB task order) depended on processes which overlapped with temporal binding (e.g. causal perception), but sensory attenuation scores later in the experiment depended on different processes (e.g. habituation or operant conditioning). Finally, there was a potentially interesting correlation between individuals' scores on the Locus of Control and Magical Ideation questionnaires, indicating that individuals with a strong internal locus of control were less likely to engage in magical thinking. However, neither scale correlated with the Likert scale SoA ratings nor with the implicit measures of SoA. In summary, our results suggest that the putative implicit and explicit measures of the SoA investigated in the present study tap into different types of self-attribution processes.

It should be noted that we only tested participants within a single, fairly constrained task environment. It is possible that sensory attenuation, temporal binding, and explicit self-attributions might be more strongly correlated in other contexts or sensory modalities. Furthermore, there are multiple ways of measuring both temporal binding and sensory attenuation and our results may have been different had we used other methods. For example, temporal binding can be measured using the interval reproduction method reported here, but is also commonly assessed using the Libet clock method. As noted in the introduction, each approach has its strength and weaknesses. The Libet clock method allows experimenters to differentiate between shifts in the perceived time of actions and their effects. However, as noted by Humphreys and Buehner [25], shifts in event boundaries could theoretically occur independently of changes in the representation of the temporal interval separating the two events. A possible explanation of our results is that temporal binding effects revealed by interval reproduction are driven by the perception of a causal relationship between two events, which do not necessarily involve intentionality or agency. It remains possible that self-agency modulates the perception of event *boundaries*, but does not modulate the perception of temporal intervals per se. In any case, it would be interesting to investigate whether explicit agency ratings are more strongly correlated with temporal binding as measured via the Libet clock procedure. Similarly, sensory attenuation can be tested using the PSE method reported here, but can also be quantified as a shift in perceptual sensitivity (d-prime) [54], or using electrophysiological indices [39], [40], [43]. It would be worthwhile to investigate how these alternative measures of sensory attenuation relate to SoA, and to each other.

A further caveat to our results is that there was arguably little ambiguity about participants' status as causal agents in the operant condition of either task (although there was still considerable variability across individuals’ Likert scale ratings). We cannot rule out the possibility that the causal relationship between the key presses and the tones seemed so obvious that the processes which drive sensory attenuation and temporal binding (e.g. predictive forward models in the motor system) were largely irrelevant to the explicit agency judgments. In this case, explicit agency judgments might be more strongly correlated with temporal binding and sensory attenuation scores under conditions of greater uncertainty (e.g. a degraded stimulus).

Importantly, this study was not designed to specify mechanisms underlying the varying measures of SoA. This would require adding further conditions in order to compare the necessary and sufficient conditions for temporal binding, sensory attenuation, and explicit self-attributions. This would be an ambitious if worthwhile project, but is beyond the scope of the present study. Previous findings do suggest that temporal binding and sensory attenuation rely on an integration of multiple cues which partially overlaps with those responsible for the SoA [7] [8]. Practically speaking, however, the relatively weak correlations across measures in the present study do not support the use of sensory attenuation (as assessed using the PSE method) or temporal binding (as assessed using temporal interval reproduction) as proxy measures for explicit self-attributions.

In conclusion, our results suggest that certain implicit measures of SoA tap into different processes than explicit self-attributions. Despite notable limitations, we believe the present study represents an important early step towards validating or invalidating alternatives to explicit self-attributions in studies of the SoA.

## Supporting Information

Table S1 **Data for computing correlation tables.**
(CSV)

Table S2 **Data for ANOVA on temporal binding.**
(CSV)

Table S3 **Data for ANOVA on sensory attenuation.**
(CSV)
